# Continuous arterial and venous glucose monitoring by quenched chemical fluorescence in ICU patients after cardiac surgery

**DOI:** 10.1186/cc12399

**Published:** 2013-03-19

**Authors:** S Bird, L Macken, O Flower, F Bass, N Hammond, S Webb, N Kennedy, A Baker, E Yarad, C Chau, M Librande, P Strasma, S Finfer

**Affiliations:** 1 Royal North Shore Hospital, Sydney, Australia; 2 Glumetrics, Irvine, CA, USA

## Introduction

Continuous glucose monitoring (CGM) in ICUs has the potential to improve patient safety outcomes. The GluCath Intravascular CGM System uses a novel quenched chemical fluorescence sensing mechanism to measure glucose concentration in venous or arterial blood (BG). This is the first report of this system deployed for 48 hours in both arteries and veins of ICU patients.

## Methods

This ongoing clinical study evaluates up to two sensors per subject in 20 patients undergoing cardiac surgery. An arterial sensor is deployed via a standard 20 G radial artery catheter inserted for routine care and an optional venous sensor is deployed percutaneously in an upper arm vein. Data are presented from the first five patients. Outcome measures are qualitative (ease of use, workflow fit) and quantitative (accuracy vs. reference analyzer). Sensors were inserted shortly after ICU admission, with ultrasound guidance for venous sensor insertion. *In vivo *calibration was performed at 1 and 2 hours, then each morning. Glucose values were recorded every 10 seconds by the system. Hourly arterial reference samples were analyzed via Radiometer ABL 800 Flex Blood Gas Analyzer (BGA).

## Results

**Figure 1:**
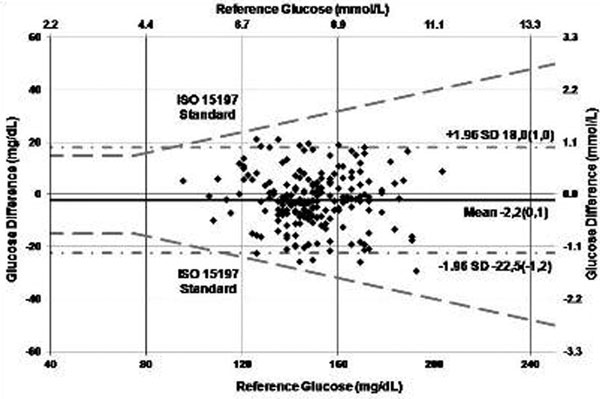
**Arterial sensor ISO-modified Bland-Altman plot**.

Arterial sensors were successfully deployed in all five patients and did not interfere with clinical care, blood pressure monitoring or sampling. One arterial catheter failed resulting in sensor removal at 36 hours. The venous sensor was deployed in three patients, but removed from two patients due to thrombosis identified during surveillance ultrasound examinations. A total of 202 reference BG samples ranging from 5.3 to 11.3 mmol/l were collected. Precision between arterial and venous sensors (*n *= 2) was 9.3% CV. Arterial sensor accuracy compared with BGA was 5.5% MARD. One hundred percent (202/202) of arterial sensor measurements met ISO 15197 criteria (within =/-20% of reference measurements if BG = 4.2 mmol/l; Figure 1).

## Conclusion

The GluCath System measured glucose concentration continuously in cardiac surgery ICU patients. Arterial catheter deployment did not appear to compromise line function or patient care. Percutaneous venous deployment was feasible, but may be associated with risk of local venous thrombosis.

